# Biochanin A Modulates Cell Viability, Invasion, and Growth Promoting Signaling Pathways in HER-2-Positive Breast Cancer Cells

**DOI:** 10.1155/2009/121458

**Published:** 2010-02-11

**Authors:** Vikas Sehdev, James C. K. Lai, Alok Bhushan

**Affiliations:** Department of Biomedical and Pharmaceutical Sciences, College of Pharmacy Idaho, Biomedical Research Institute, Idaho State University, Pocatello, ID 83209, USA

## Abstract

Overexpression of HER-2 receptor is associated with poor prognosis and aggressive forms of breast cancer. Scientific literature indicates a preventive role of isoflavones in cancer. Since activation of HER-2 receptor initiates growth-promoting events in cancer cells, we studied the effect of biochanin A (an isoflavone) on associated signaling events like receptor activation, downstream signaling, and invasive pathways. HER-2-positive SK-BR-3 breast cancer cells, MCF-10A normal breast epithelial cells, and NIH-3T3 normal fibroblast cells were treated with biochanin A (2–100 *μ*M) for 72 hours. Subsequently cell viability assay, western blotting and zymography were carried out. The data indicate that biochanin A inhibits cell viability, signaling pathways, and invasive enzyme expression and activity in SK-BR-3 cancer cells. Biochanin A did not inhibit MCF-10A and NIH-3T3 cell viability. Therefore, biochanin A could be a unique natural anticancer agent which can selectively target cancer cells and inhibit multiple signaling pathways in HER-2-positive breast cancer cells.

## 1. Introduction

Breast cancer is the most frequently diagnosed and second-most morbid form of cancer afflicting women in the USA. [1]. Breast cancer is a heterogeneous disease with various subtypes exhibiting differential susceptibility to anticancer drugs. Therefore, therapy targeting the specific breast cancer subtype is recommended for effective treatment [2, 3]. During the normal course of cellular life-cycle cells grow, differentiate, and die. These three fundamental stages of the cellular life-cycle are highly regulated in normal cells whereas uncontrolled growth, dedifferentiated morphology, and resistance to death are the hallmarks of cancer cells [4, 5]. Breast cancer cells also exhibit deregulated signaling pathways that promote uncontrolled growth, impart resistance to cell death, and induce invasion into the surrounding tissues [6].

Breast cancer cell proliferation is known to be regulated by two main families of receptors: (1) hormonal receptors (estrogen receptor or ER and progesterone receptor or PR) and (2) receptor tyrosine kinases or RTKs (like human epidermal growth factor receptors or HERs) [7]. Based on the receptors present, tumors of the breast can be broadly classified into (a) luminal A subtype (ER and/or PR-, HER-2-), (b) luminal B subtype (ER+ and/or PR+, sometimes HER-2+), (c) HER-2/ER- subtype (HER-2+ and ER-/PR-), and (c) basal subtype (ER-/PR-/HER-2-) breast cancer [8]. HER-2/ER- and basal subtypes of breast cancer are aggressive, resistant to antihormonal therapy and have poor prognosis as compared to luminal A and luminal B subtypes of breast cancer [8, 9]. The hormone receptor negative breast cancer subtypes (HER-2/ER- and basal) respond to chemotherapeutic drugs; however, HER-2/ER- is indicated to be relatively resistant to chemotherapeutic drugs [10]. 

The above observations indicate that HER-2 overexpressing breast tumors are difficult to treat. HER-2 (also known as EGFR2/*erb*B-2) receptor is a proto-oncogene which belongs to the HER-family of RTKs [11]. The HER-family of RTKs initiates transient potent downstream signaling events which regulate growth, viability, and morphogenesis of normal breast epithelial cells [12]. Overexpression and/or amplification of HER-2 proto-oncogene is observed in about 30% of all the breast cancer cases [13]. Since HER-2 receptor is the most favored dimerizing partner for other HER-family of receptors, elevated levels of HER-2 receptor activity can mediate cellular proliferation [11]. Therefore, HER-2 receptor and its downstream signaling pathways are important therapeutic targets for breast cancer therapy.

Various genetic and epigenetic factors influence breast cancer incidence by modulating expression and/or activity of proto-oncogenes and tumor suppressor genes [14, 15]. Genetic factors account for about 5%–10% of breast cancer cases whereas epigenetic factors like diet play a major role in breast cancer incidence [16, 17]. Population-based epidemiological studies on an international scale indicate that breast cancer incidence and mortality rates in Asian populations are lower than that in the US population [18]. When Asians migrate to USA, their incidence for breast cancer increases over a period of several generations and becomes similar to that of the American population [17, 19]. Epigenetic factors like change in food habits have been implicated for increase in breast cancer incidence [17]. Doll and Peto [20] and Willett [21, 22] have reported that about 32% of cancer cases could be avoided by dietary modifications [20–22].

Consumption of diets rich in fruits, green leafy vegetables, and soy-based products has been correlated with low breast cancer incidence in certain subsets of populations [23–25]. Rich in isoflavones, soy, soy-based products, chick peas, and lentils constitutes a major source of protein in Asian diets. Elevated serum levels of isoflavones due to consumption of isoflavone-rich dietary ingredients has been correlated with a reduced risk of breast cancer among women in China, Japan and The Netherland [26–28].

Isoflavones are generally well tolerated and have not exhibited toxicity in humans [29]. Genistein is one of the most widely studied isoflavones. Genistein is indicated to inhibit growth of ER+ and HER-2+ breast cancer cells, delay onset of spontaneous mammary tumors in MMTV-neu mouse model, induce cellular differentiation, and inhibit cell cycle progression in ER+ and HER-2+ breast cancer cells [30–35]. However, genistein has shown mutagenic activity in mouse lymphoma assay and Chinese hamster V79 cells [36, 37]. Genistein-mediated irreversible inhibition of Topoisomerase-II activity could result in the indicated mutagenic effect [36]. Biochanin A, an isoflavone found in red clover, has been shown to prevent genistein's mutagenic effect in Chinese hamster V79 cells [38]. Additionally, in vitro experiments with HMECs (primary human mammary epithelia cells), MCF-12A (a nontumorigenic immortalized breast epithelial cell line), and MCF7 (ER-positive breast cancer cell line) cells indicate that biochanin A primarily induces expression of favorable tumor suppressor genes in HMEC and MCF 12A cell lines [39]. Since biochanin A is better tolerated and has favorable protein expression effects as compared to genistein, we studied the effect of biochanin A on pathways critical for cancer cell growth, viability, and metastasis. Our previous data indicate that biochanin A can inhibit growth and survival regulating pathways in oral squamous cell carcinoma cells [40]. The goal of this study was to determine the effect of biochanin A treatment on pathways regulating cellular viability, invasion, and growth in HER-2-positive (HER-2+) breast cancer cells. Our hypothesis is that “biochanin A inhibits HER-2 receptor activation, cytoplasmic signaling pathways, and transcriptional factors associated with growth, viability, and invasion of HER-2+ SK-BR-3 breast cancer cells”.

## 2. Materials and Methods

### 2.1. Cell Culture and Cell Lines

SK-BR-3, MCF-10A, and NIH-3T3 cell lines were obtained from American Type Culture Collection (ATCC). SK-BR-3 breast cancer cell line was maintained as a monolayer culture in RPMI-1640 cell culture medium supplemented with 10% (v/v) fetal bovine serum (FBS). The SK-BR-3 breast cancer cell line is ER-, PR-, and overexpresses HER-2 receptor protein [41]. The MCF-10A-immortalized breast epithelial cell line was cultured in DMEM/F12 (1 : 1) cell culture media supplemented with 5% (v/v) horse serum, insulin (10 *μ*g/mL), epidermal growth factor (20 ng/mL), hydrocortisone (0.5 *μ*g/mL), cholera toxin (100 ng/mL) and penicillin-streptomycin (100 *μ*g/mL each) according to the protocol suggested by Zientek-Targosz et al. [42] and Debnath et al. [43]. MCF-10A breast epithelial cells are devoid of HER-2 receptor protein [44]. The NIH-3T3 normal fibroblast cell line was cultured in DMEM cell culture media supplement with 10% (v/v) FBS.

### 2.2. Pharmacological Treatments

Biochanin A (D2016) stock solutions were prepared with dimethy sulfoxide or DMSO (D4540) as the vehicle. Individual stock solutions were prepared for each biochanin A final treatment concentration of 2, 5, 10, 20, 50, 60, 70, 80, 90, and 100 *μ*M so that the final volume of DMSO was same in all the treatments. Cells were treated once with biochanin A for 72 hours.

### 2.3. Antibodies

The following antibodies were procured from Santa Cruz Biotechnology (Santa Cruz, CA) and Cell Signaling Technology Inc. (Danvers, MA): Akt (SC-8312), p-Akt (SC-16646-R), *β*-Actin (SC-47778), Erk (SC-094), p-Erk (SC-7976-R), HER-2 (SC-284), p-HER-2 (SC-12352-R), mTOR (SC-8319), p-mTOR (2971), NF*κ*B (SC-109), and MT-MMP-1 (SC-12367).

### 2.4. MTT Cell Viability Assay

SK-BR-3/NIH-3T3 and MCF-10A cells were seeded at 2000 and 5000 cells/well, respectively, into 96-well plates. The plated cells were treated with biochanin A (2–100 *μ*M) and incubated for 72 hours at 37°C and 5% CO_2_ level in an incubator (NuAire Inc., Plymouth, MN). Subsequently, 20 *μ*L of MTT reagent (5 mg/mL in phosphate buffered saline pH 7.2 or PBS) was added into each well and the plates were incubated for 4 hours. The violet colored formazan crystals formed in each well were dissolved in 50 *μ*L of dimethyl sulfoxide (DMSO). The absorbance of dissolved formazan crystals was measured at 540 nm in a plate-reader (Bio-Tek PowerWave, BioTek Instruments, Inc., Winooski, VT). The intensity of the signal is a measure of overall cellular metabolic activity and an indicator of viable cells present at the end of the treatment.

### 2.5. Western Blot Analysis

SK-BR-3 breast cancer cells were treated with biochanin A (2–50 *μ*M) for 72 hours and cell lysates prepared using cell lysis buffer (containing 1% (v/v) Triton X-100, 10 mM Tris base pH 7.6, 5 mM EDTA, 50 mM sodium chloride, 30 mM sodium pyrophosphate, 50 mM sodium fluoride, 0.1% (w/v) sodium azide, 50 mM phenyl methyl sulfonyl fluoride, 0.5 mg/mL aprotinin, 2.5 mg/mL leupeptin, and 100 mM sodium orthovandate in distilled water at pH 7.6). Protein concentration was determined using BioRad reagents (Bradford dye) with photometric analysis at 570 nm. Twenty five microgram of cell lysate proteins and 10 *μ*L of protein ladder (Boston Bioproducts, Worcester, MA) were separated by sodium dodecyl sulfate polyacrylamide gel electrophoresis (SDS-PAGE) and transferred on to polyvinylidene fluoride membrane (PVDF) (Millipore, Bedford, MA). The membranes were incubated with a primary antibody against total and phosphorylated proteins (HER-2, mTOR, Erk, Akt, NF*κ*B or MT-MMP1) (Santa Cruz Biotechnology, Santa Cruz, CA and Cell Signaling, Boston, MA) at 1 : 500 dilution in 5% (w/v) bovine serum albumin (BSA) and 0.01% (w/v) sodium azide in double distilled water. The membrane was then treated with horseradish peroxidase-linked secondary antibody at 1 : 5000 dilution in 5% milk solution in Tris buffered saline containing 0.1% (v/v) Tween-20 (TBST) for 45 minutes at room temperature. The blots were then developed by using chemiluminescence reagents (Pierce Biotechnology, Rockford, IL), according to the manufacturer's protocol and autoradiographed. The blots were scanned to determine the relative intensities of the protein bands using UNSCAN-IT software version 6.1 (Silk Scientific, Orem, UT). Western blot analysis using an antibody against *β*-Actin (Santa Cruz Biotechnology, Santa Cruz, CA) was carried out and its relative intensity was determined by UNSCAN-IT. The *β*-Actin band data were used to normalize the band intensities of protein loaded into each lane of the separation gel.

### 2.6. Gelatin Zymography

3 × 10^6^ SK-BR-3 breast cancer cells were seeded in a T-75 tissue culture flask in RPMI-1640 (10% (v/v) FBS) for 24 hours. The cells were then treated with biochanin A (20 and 50 *μ*M) for 72 hours in serum free RPMI-1640. The serum free conditioned medium was collected after 72 hours of treatment and centrifuged (1000 rpm, 4°C, 10 minutes) to remove any floating cellular debris. The remaining supernatant (25 mL) media was freeze dried overnight and reconstituted with 2.5 mL of double distilled water. The samples were electrophoresed on commercially available 10% (w/v) SDS polyacrylamide gels embedded with gelatin (BioRad, Hercules, CA). After electrophoresis, the gels were washed with renaturation buffer (2.5% (v/v) Triton X-100 solution for 1 hour to remove SDS. Gels were then incubated over night at 37°C in the development buffer (50 mM Tris-base, 200 mM NaCl, 5 mM CaCl_2_, and 0.02% (w/v) Brij-35 at pH 7.6). The gels were stained with the staining solution (40% (v/v) methanol, 10% (v/v) acetic acid, and 0.5% (w/v) coomassie blue and water) for 1 hour and then destained using a destaining solution (40% (v/v) methanol, 10% (v/v) acetic acid, and 50% (v/v) water). Gelatinolytic activity was identified as transparent (digested) bands on a blue background. The 72 kDa band corresponds to MMP-2 and the 92 kDa band corresponds to MMP-9.

### 2.7. Statistical Analysis

Data were expressed as arithmetic means ± standard error of mean. The number of “*n*” in the figure legends represents the number of independent experiments. One-way analysis of variance (ANOVA) with Tu-Key post hoc analysis was used to show statistical difference between control groups and biochanin A treatment. All statistical analyses were carried out using Kaleidagraph 4.0 (Synergy software, PA), and *P* < .05 was considered as statistically significant.

## 3. Results

Elevated HER-2 receptor expression and/or activity is observed in about 30% of breast cancer cases [13]. Clinically, HER-2+ breast tumor status has been associated with absence of ER/PR and poor overall therapeutic outcome [45]. Since the SK-BR-3 breast cancer cells overexpress HER-2 receptor protein and lack ER and PR, we used SK-BR-3 breast cancer cell line to study the effect of biochanin A on HER-2/ER- subtype of breast cancer [41]. The MCF-10A-immortalized breast epithelial cell line was used as a normal control breast epithelial cell line because it exhibits phenotypical characteristics similar to those of normal breast epithelial cells. The MCF-10A cells are nontumorigenic, require hormones and growth factors for growth, form three-dimensional structures in collagen, exhibit anchorage dependent growth, and respond to growth regulatory mechanisms [43, 46, 47]. In this study we determined the effect of biochanin A on cell viability, signaling, and invasive pathways in SK-BR-3 breast cancer cells. We also studied inhibition of MCF-10A and NIH-3T3 cell viability with biochanin A treatment.

### 3.1. Effect of Biochanin A on SK-BR-3, MCF-10A, and NIH-3T3 Cell Viability

**Figure 1:**
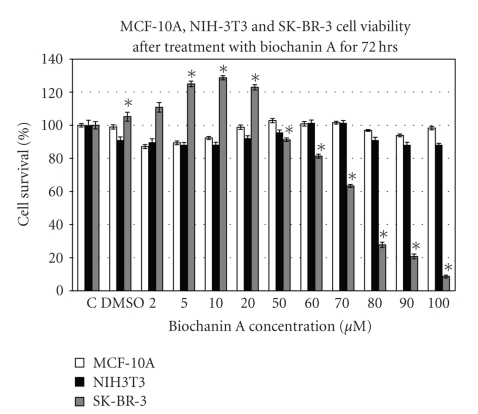
Effect of biochanin A on MCF-10A, NIH-3T3, and SK-BR-3 cell viability. SK-BR-3/NIH-3T3 and MCF-10A cells were seeded at 2000 and 5000 cells/well in a 96-well plate. After 8 hours of cell adhesion period, the cells were treated with biochanin A (2–100 *μ*M) for 72 hours. The data indicate a biphasic effect of biochanin A on SK-BR-3 breast cancer cell viability. Biochanin A did not inhibit MCF-10A and NIH-3T3 cell viability. *C: control or no treatment; DMSO: vehicle* (Statistical analysis: One-way Anova, *n* = 3, *P < .05).

The cell viability assay data (Figure 1) indicate that treatment of SK-BR-3 breast cancer cells with biochanin A (2–100 *μ*M) has a biphasic effect on cancer cell viability. Treatment with 5–20 *μ*M biochanin A induces increased SK-BR-3 breast cancer cell viability whereas treatment with 50–100 *μ*M biochanin A induces a dose-dependent inhibition of cell viability. In contrast with SK-BR-3 breast cancer cell line, treatment of MCF-10A and NIH-3T3 cell lines with biochanin A (2–100 *μ*M) did not inhibit MCF-10A and NIH-3T3 cell viability (Figure 1).

Current synthesis of literature suggests that HER-family of receptors initiate intracellular signaling events that play an important role in the development, proliferation, and spread of HER-2/ER- subtype of breast cancer. According to our cell viability assay data (Figure 1), biochanin A mediated biphasic effect on SK-BR-3 breast cancer cell viability lies within 2–50 *μ*M concentration range. Therefore, we determined the effect of biochanin A on SK-BR-3 breast cancer cell signaling by treating the cells with 2–50 *μ*M biochanin A.

### 3.2. Biochanin A Inhibits HER-2 Receptor Activation

**Figure 2:**
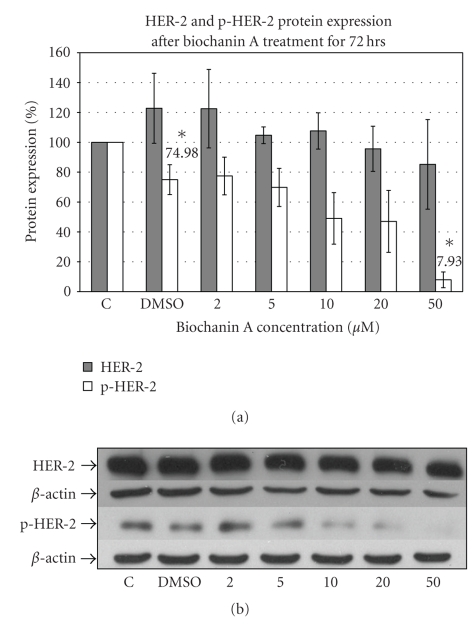
Effect of biochanin A on HER-2 and p-HER-2 protein expression. (a) SK-BR-3 breast cancer cells were seeded in a T-75 flask in RPMI-1640 cell culture media (10% FBS). Following 72 hours of treatment with biochanin A (2–50 *μ*M) HER-2 and p-HER-2 protein expression was evaluated. The data indicate reduced HER-2 phosphorylation with 50 *μ*M biochanin A (7.93 ± 5.27; *P* < .05). *C: control or no treatment; DMSO: vehicle* (Statistical analysis: One-way Anova, *n* = 3, *P < .05). (b) Representative blot for HER-2 and p-HER-2 protein expression.

A comprehensive review by Ross et al. in 2003 of 80 published clinical studies suggested a strong correlation between HER-2 overexpression and poor breast cancer patient viability rates [45]. Increased level of HER-2 receptor protein has also been associated with aggressive and malignant form of breast cancer with high morbidity rates [45]. Western blot analysis was carried out to determine changes in levels of total or phosphorylated HER-2 protein. Western blotting data (Figure 2) with HER-2 specific antibody indicate inhibition of HER-2 receptor phosphorylation with 50 *μ*M (7.93 ± 5.27; *P* < .05) biochanin A treatment.

Reduced HER-2 receptor activation with biochanin A could further result in inhibition of downstream signaling pathways regulating growth, viability, and invasion.

### 3.3. Biochanin A Inhibits Erk1/2 (MAPK) Activation

**Figure 3:**
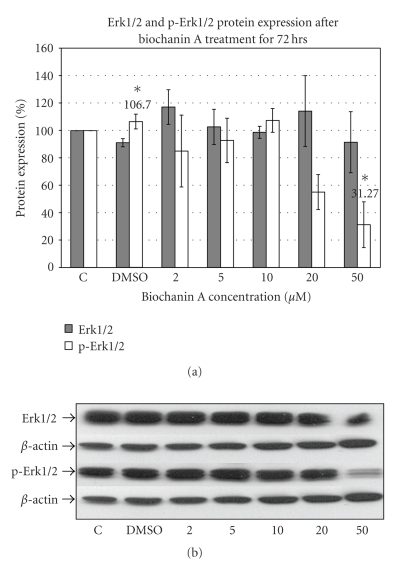
Effect of biochanin A on Erk1/2 and p-Erk1/2 protein expression. (a) SK-BR-3 breast cancer cells were seeded in a T-75 flask in RPMI-1640 cell culture media (10% FBS). Following 72 hours of treatment with biochanin A (2–50 *μ*M) Erk1/2 and p-Erk1/2 protein expression was evaluated. The data indicate reduced Erk1/2 phosphorylation at 50 *μ*M biochanin A (31.27 ± 16.71; *P* < .05). *C: control or no treatment; DMSO: vehicle* (Statistical analysis: One-way Anova, *n* = 3, *P < .05). (b) Representative blot for Erk1/2 and p-Erk1/2 protein expression.

The Erk1/2 (extracellular signal regulated kinase) pathway is one of the major downstream signaling pathways associated with potent mitogenic effect [48]. Western blotting data (Figure 3) with Erk1/2 specific antibody indicate suppression of Erk1/2 phosphorylation with 50 *μ*M (31.27 ± 16.71; *P* < .05) biochanin A treatment in SK-BR-3 breast cancer cells.

### 3.4. Biochanin A Inhibits Akt and mTOR Activation

**Figure 4:**
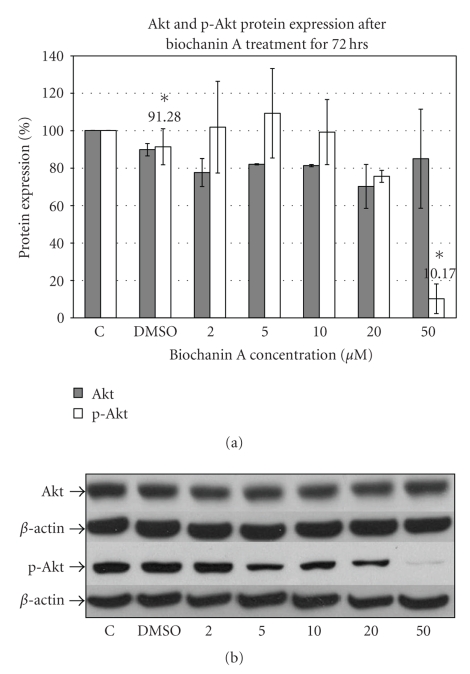
Effect of biochanin A on Akt and p-Akt protein expression. (a) SK-BR-3 breast cancer cells were seeded in a T-75 flask in RPMI-1640 cell culture media (10% FBS). Following 72 hours of treatment with biochanin A (2–50 *μ*M) Akt and p-Akt protein expression was evaluated. The data indicate reduced Akt phosphorylation at 50 *μ*M (10.17 ± 7.89; *P* < .05) biochanin A. *C: control or no treatment; DMSO: vehicle* (Statistical analysis: One-way Anova, *n* = 3, *P < .05). (b) Representative blot for Akt and p-Akt protein expression.

The Akt pathway is another major downstream signaling pathway associated with cellular growth and viability [49]. Western blotting data (Figure 4) with Akt specific antibody indicate inhibition of Akt phosphorylation with 50 *μ*M (10.17 ± 7.89; *P* < .05) biochanin A treatment in SK-BR-3 breast cancer cells.

**Figure 5:**
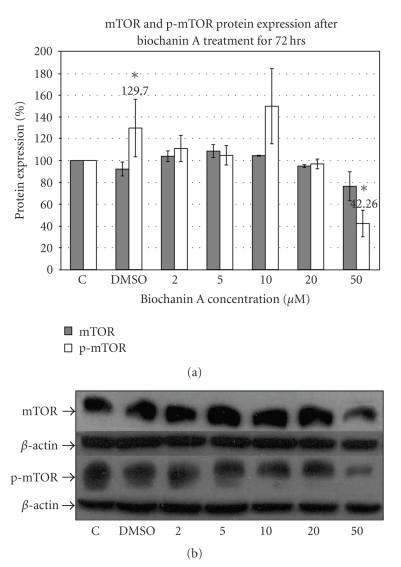
Effect of biochanin A on mTOR and p-mTOR protein expression. (a) SK-BR-3 breast cancer cells were seeded in a T-75 flask in RPMI-1640 cell culture media (10% FBS). Following 72 hours of treatment with biochanin A (2–50 *μ*M) mTOR and p-mTOR protein expression was evaluated. The data indicate reduced mTOR phosphorylation at 50 *μ*M biochanin A (42.26 ± 12.18; *P* < .05). *C: control or no treatment; DMSO: vehicle* (Statistical analysis: One-way Anova, *n* = 3, *P < .05). (b) Representative blot for mTOR and p-mTOR protein expression.

Phosphorylated Akt can subsequently activate the downstream mTOR signaling pathway which regulates cell cycle progession [49, 50]. The mTOR protein expression data (Figure 5) with phospho-specific mTOR antibody indicate inhibition of mTOR phosphorylation with 50 *μ*M biochanin A treatment (42.26 ± 12.18; *P* < .05) in SK-BR-3 breast cancer cells.

Erk1/2, Akt, and mTOR signaling pathways can subsequently modulate effector transcriptional factor expression and/or activity.

### 3.5. Biochanin A Inhibits NF*κ*B Transcriptional Factor Expression

**Figure 6:**
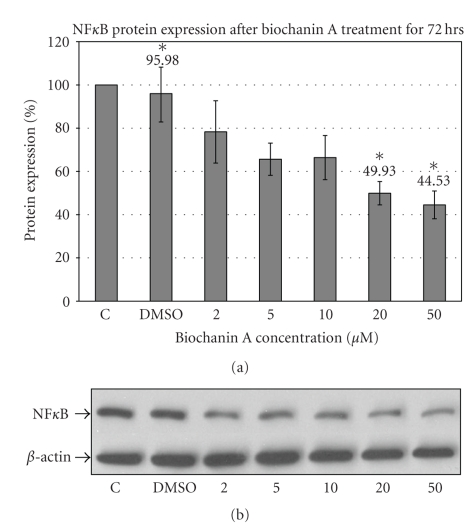
Effect of biochanin A on NF*κ*B protein expression. (a) SK-BR-3 breast cancer cells were seeded in a T-75 flask in RPMI-1640 cell culture media (10% FBS). Following 72 hours of treatment with biochanin A (2–50 *μ*M) NF*κ*B protein expression was evaluated. The data indicate reduced NF*κ*B expression at 20 and 50 *μ*M biochanin A (Biochanin A: 20 *μ*M  → 49.93 ± 5.41; 50 *μ*M  → 44.53 ± 6.44; *P* < .05). *C: control or no treatment; DMSO: vehicle* (Statistical analysis: One-way Anova, *n* = 3, *P < .05). (b) Representative blot for NF*κ*B protein expression.

NF*κ*B is a transcriptional factor found to be constitutively active in HER-2-positive breast cancer subtype [51]. Western blotting data (Figure 6) with NF*κ*B specific antibody indicate suppression of NF*κ*B expression after treating SK-BR-3 cells with 20 (49.93 ± 5.41; *P* < .05) and 50 *μ*M (44.53 ± 6.44; *P* < .05) biochanin A (Figure 6). 

Elevated oncogenic signaling has been associated with malignant transformation of breast cancer cells. Malignant cancer cells are indicated to overexpress matrix metalloproteases (MMPs) which aid in the process of tumor cell invasion and migration.

### 3.6. Biochanin A Inhibits MMP-9 Activity and MT-MMP1 Expression

**Figure 7:**
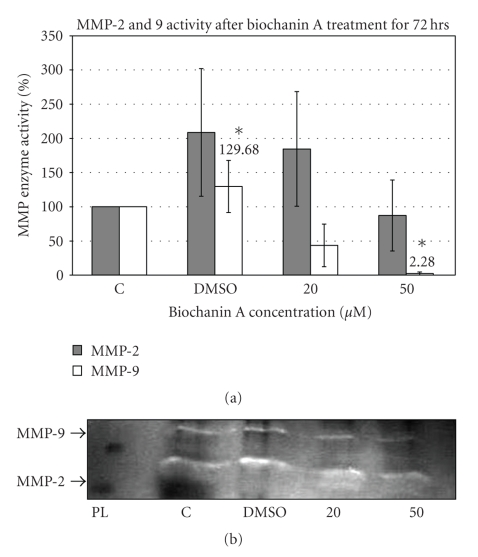
Effect of biochanin A on MMP-2 and MMP-9 activity. (a) SK-BR-3 breast cancer cells were seeded in a T-75 flask in RPMI-1640 cell culture media (10% FBS) for 24 hours. Subsequently they were treated for 72 hours with biochanin A (20 and 50 *μ*M) in serum free conditions and MMP-2 and MMP-9 gelatinase enzyme activity was evaluated. The data indicate reduced MMP-9 activity with 50 *μ*M biochanin A (2.28 ± 2.28; *P* < .05). *C: control or no treatment; DMSO: vehicle; PL: protein ladder* (Statistical analysis: One-way Anova, *n* = 3, *P < .05). (b) Representative gelatin gel for MMP-2 and MMP-9 activity.

MMPs facilitate cancer cell movement into the surrounding region by digesting the extracellular matrix (ECM) components around the tumor cells (Ref). The MMP gelatinase activity or gelatin zymography assay data (Figure 7) indicate inhibition of MMP-9 enzyme activity in SK-BR-3 cells treated with 50 *μ*M biochanin A (2.28 ± 2.28; *P* < .05).

**Figure 8:**
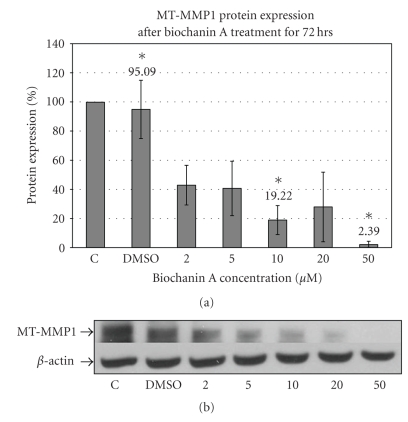
Effect of biochanin A on MT-MMP1 protein expression. (a) SK-BR-3 breast cancer cells were seeded in a T-75 flask in RPMI-1640 cell culture media (10% FBS). Following 72 hours of treatment with biochanin A (2–50 *μ*M) MT-MMP1 protein expression was evaluated. Our data indicate reduced MT-MMP-1 expression at 10 and 50 *μ*M biochanin A (Biochanin A: 10 *μ*M  → 19.22 ± 9.93; 50 *μ*M  → 2.39 ± 2.30; *P* < .05). *C: control or no treatment; DMSO: vehicle* (Statistical analysis: One-way Anova, *n* = 3, *P < .05). (b) Representative blot for MT-MMP-1 protein expression.

MT-MMP1 or MMP-14 is required for activation of matrix metalloproteases (MMPs) [52]. Western blotting data (Figure 8) with MT-MMP1 specific antibody indicate inhibition of MT-MMP1 enzyme expression in SK-BR-3 cells treated with 10 (19.22 ± 9.93; *P* < .05) and 50 *μ*M (2.39 ± 2.30; *P* < .05) biochanin A (Biochanin A: 10 *μ*M  → 19.22 ± 9.93; 50 *μ*M  → 2.39 ± 2.30; *P* < .05) (Figure 8). Biochanin A-mediated inhibition of MMP-9 enzyme activity and MT-MMP1 protein expression suggests that biochanin A can block invasion promoting signaling mechanisms in SK-BR-3 breast cancer cells.

## 4. Discussion

Uncontrolled growth, resistance to chemotherapeutic drugs, and tumor invasion are the major limiting factors preventing successful chemotherapeutic outcome. Elevated activity of HER-2 receptor plays a major role in promoting growth, viability, and invasion regulating signaling mechanisms in HER-2/ER- subtype of breast cancer [53]. HER-2 is the favored dimerizing partner for the HER-family members and an important therapeutic target for breast cancer treatment. Increase in HER-2 receptor expression has been shown to enhance bladder cancer cell sensitivity to physiological levels of isoflavones [54]; therefore, suggesting HER-2 receptor as a biochemical target for isoflavones [54]. Genistein, the most widely studied isoflavone, is indicated to inhibit receptor tyrosine kinase activity, HER-2 receptor activation, downstream signaling pathways, and delay onset of spontaneous mammary tumors in MMTV/neu mouse model [32, 34, 55]. Unlike genistein, biochanin A is indicated to be nonmutagenics and prevent genistein-induced mutagenic effect [36, 38]. Since biochanin A is a methyl derivative of genistein, we hypothesized that biochanin A inhibits HER-2 receptor activation and downstream signaling pathways associated with growth, viability, and invasion of HER-2+ breast cancer cells. We also studied the relative cytotoxic effect of biochanin A treatment on SK-BR-3 breast cancer cell and MCF-10A normal breast epithelial cell viability. 

To test our hypothesis we carried out cell viability analysis by treating SK-BR-3 and MCF-10A cells with biochanin A (2–100 *μ*M). The data (Figure 1) suggest that biochanin A selectively inhibits SK-BR-3 breast cancer cells as compared to MCF-10A normal breast epithelial and NIH-3T3 normal fibroblast cells. It also indicates that biochanin A has a biphasic effect on SK-BR-3 cell viability with increased cell viability at subapoptotic concentrations (2–20 *μ*M) and a dose dependent inhibition of cell viability at higher concentrations (50–100 *μ*M). The increased cellular viability observed with subapoptotic concentrations of isoflavones like genistein and resveratrol could be due to increased mitochondrial number and/or activity, induction of cellular differentiation, and/or cell cycle arrest in G2/M phase [56–59]. Isoflavone-induced proliferative effect on ER+ MCF-7 cells in vitro and rodent models in vivo has been attributed to their weak estrogenic activity at subapoptotic concentrations [60].

Since HER-2 receptor plays an important role in regulating growth, viability, and invasion regulating signaling mechanisms in HER-2/ER- subtype of breast cancer, we determined the effect of biochanin A on HER-2 receptor activation in SK-BR-3 breast cancer cells. Our protein expression data (Figure 2) indicate that 50 *μ*M biochanin A treatment significantly inhibits HER-2 receptor activation (7.93 ± 5.27; *P* < .05). Activated HER-2 receptor initiates growth, viability, and invasion promoting downstream signaling pathways [53]. In this study we determined the effect of biochanin A on two major downstream signaling pathways which could be activated by HER-2 receptor: (1) the Erk1/2 pathway, and (2) the Akt/mTOR pathway. 

Constitutive over-activity of the Erk1/2 pathway has been observed in both breast cancer tissue and cell lines as compared to normal tissues and cells [48, 61, 62]. Moreover, increased activity of the Erk1/2 pathway is associated with drug resistance, cellular proliferation, and increased metastasis in breast cancer cells [63]. The Akt pathway is a downstream signaling pathway associated with resistance to drug induced apoptosis and deregulation of endogenous cell death mechanisms [49]. Activated Akt can initiate signaling pathways which can lead to cellular proliferation and resistance to cell death. An important downstream signaling pathway activated by Akt is the mTOR pathway. Increased phosphorylation of mTOR is associated with increased protein synthesis, a prerequisite for cellular proliferation [49]. Our protein expression data (Figures 3, 4, and 5) suggest that biochanin A at 50 *μ*M can inhibit activation of the Erk1/2 (31.27 ± 16.71; *P* < .05), Akt (10.17 ± 7.89; *P* < .05), and mTOR (42.26 ± 12.18; *P* < .05) pathways.

Activation of cytoplasmic signaling pathways (Erk1/2, Akt, and mTOR) results in activation of transcriptional factors which mediate expression of specific proteins. HER-2-activated Akt pathway has been shown to regulate NF*κ*B transcriptional factor expression in breast cancer cells [64]. Elevated level of NF*κ*B can promote cellular growth, viability and malignant transformation [51]. High levels of NF*κ*B expression have been correlated with HER-2/ER-subtype of breast cancer. The NF*κ*B protein expression data (Figure 6) indicate that biochanin A (20 and 50 *μ*M) can inhibit NF*κ*B (Biochanin A: 20 *μ*M  → 49.93 ± 5.41; 50 *μ*M  → 44.53 ± 6.44; *P* < .05) expression which is consistent with HER-2/Akt-mediated regulation of NF*κ*B expression.

Matrix Metalloproteases (MMPs) are protein-digesting enzymes with the primary function of breaking down components (extracellular matrix, ECM) surrounding the tumor cells. This activity of MMPs is employed by cancer cells to invade surrounding tissue and metastasize. Elevated expression of MMP-2 and MMP-9 is positively correlated with HER-2 overexpression in mammary tumors [65]. The MMP-2 and MMP-9 gelatin zymography assay data (Figure 7) indicate inhibition of MMP-9 enzyme activity in SK-BR-3 cells treated with biochanin A.

MT-MMP1 or MMP-14 is required for activation of matrix metalloproteases (MMPs). MT-MMP1 is a cell membrane localized protease that cleaves the proenzyme or pro-MMP into its ECM-digesting active enzyme or MMP state. The activated MMP can then digest its specific substrate(s) within the ECM and release growth factors and cytokines for the growth of invading cancer cells. The MT-MMP1 protein expression data (Figure 8) indicate that biochanin A can inhibit MT-MMP1 protein expression in SK-BR-3 cells.

The studies presented in this report indicate that biochanin A can inhibit multiple signaling pathways associated with growth, survival, and invasion in HER-2+ breast cancer cells at supraphysiological concentrations. Previous in vitro and in vivo (xenograft) studies with MCF-7 breast cancer cells and biochanin A indicate that, compared to in vitro concentrations, biochanin A can inhibit in vivo tumor growth at much lower plasma concentrations [66, 67]. Soy meal diet has also been shown to delay spontaneous mammary tumor development in MMTV/neu mouse model [68]. This could be due to preferential accumulation of biochanin A and/or potent biochanin A metabolites (generated during in vivo metabolism) in mammary tissue. Additionally, the cell viability data (Figure 1) indicate that biochanin A treatment (50–100 *μ*M) selectively inhibits SK-BR-3 breast cancer cell viability without affecting MCF-10A normal breast epithelial cell and NIH-3T3 normal fibroblast cell viability. Further characterization of the effect of biochanin A on cell cycle progression and regulation in SK-BR-3, MCF-10A, and NIH-3T3 cells will improve our mechanistic understanding of the selective inhibitory effect of biochanin A on SK-BR-3 breast cancer cell viability.

## 5. Conclusion

**Figure 9:**
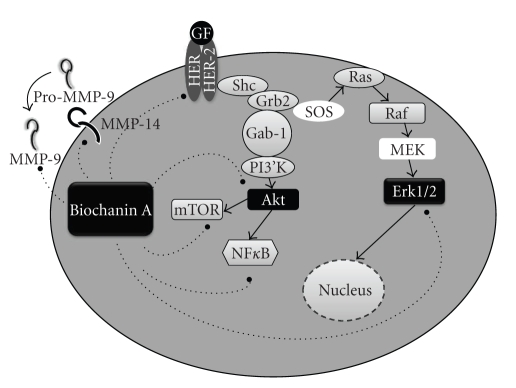
Pathways inhibited by biochanin A in HER-2+ SK-BR-3 breast cancer cells. The protein expression data indicate that biochanin A treatment inhibits growth (p-HER-2, p-Erk), survival (Akt, mTOR, and NF*κ*B), and invasion (MMP-9 and MMP-14) promoting pathways in SK-BR-3 cells.

The HER-2-positive subtype of breast cancer has an aggressive malignant phenotype due to HER-2 signaling cascade mediated rapid growth, drug resistance, and distant metastasis. This report indicates that biochanin A, a dietary isoflavone, can inhibit HER-2 receptor activation, downstream signaling pathways (Erk1/2, Akt, and mTOR), and NF*κ*B transcription factor expression. Apart from inhibiting the signaling pathways, biochanin A also inhibits invasive enzyme activity by suppressing MMP-9 protease activity and MT-MMP1 protein expression, respectively. The data (as summarized in Figure 9) suggest that biochanin A is a unique natural compound which selectively targets HER-2+ SK-BR-3 breast cancer cells and inhibits multiple deregulated mechanisms associated with malignant transformation. Therefore, biochanin A should be studied further to determine if it can be used with other conventional chemotherapeutic drugs and/or with HER-2 targeted anticancer drugs for better therapeutic outcome of patients suffering from HER-2/ER-subtype of breast cancer.
